# Should Waste Separation Be Mandatory? A Study on Public’s Response to the Policies in China

**DOI:** 10.3390/ijerph17124539

**Published:** 2020-06-24

**Authors:** Jing Hou, Yujing Jin, Feiyu Chen

**Affiliations:** 1 Business School, Jiangsu Normal University, No.101 Shanghai Road, Xuzhou 221116, China; houjing@jsnu.edu.cn; 2 School of Economics and Management, China University of Mining and Technology, No.1 Daxue Road, Xuzhou 221116, China; jinyujingcumt@163.com

**Keywords:** waste separation policy, public’s response, rigid, flexible

## Abstract

The implementation of effective waste separation policy is an important pathway to guide the public to actively participate in the waste separation action. This study focused on exploring the Chinese public’s response to the rigid and flexible waste separation policies from the perspectives of understanding, willingness to support, and willingness to implement. We used a big data mining technique to obtain 10,057 entries of the Chinese public’s response to the mandatory waste separation policy. The results showed that “public’s understanding–support willingness-implement willingness” regarding mandatory waste separation policy was characterized by a U-shaped response. Specifically, the public’s understanding and willingness to implement the rigid waste separation policy were relatively high in the short term, but their willingness to support this policy was relatively low and became increasingly low over time. Particularly, “troublesome” implementation was deemed to the main reason for the public’s low willingness to support the rigid waste separation policy. In addition, we further obtained the sample data of the Chinese public regarding the flexible waste separation policy through the situational survey. Contrary to the response characteristics of mandatory waste separation policy, the results showed that “public’s understanding-support willingness-implement willingness” regarding flexible waste separation policy was characterized by an inverted U-shaped response, and the Chinese public showed more positive sentiment regarding the willingness to support and implement. The results have important implications for guiding the public to actively participate in the waste separation action.

## 1. Introduction

The volume of domestic waste removal in Chinese cities has increased rapidly in the last decade (NBSPRC, 2019) [1], and a large number of cities fall into the dilemma of “waste siege” [2]. In response to this dilemma, Chinese government started the pilot work of urban domestic waste separation and collection in 2000 [3], attempting to guide the public’s active waste separation behavior. In 2017, the Chinese government first proposed to make it mandatory for urban residents to sort their domestic waste; in July 2019, Shanghai took the lead in formulating and implementing the mandatory waste separation policy; henceforth, China began to enter the era of rigid waste separation management. Compared with the previous waste separation policy, the rigid waste separation policy emphasizes the legal obligations of citizens and sets corresponding penalties for violations of waste separation regulations [4]. Such rigid management has indeed achieved certain results in the short term. By the end of October 2019, there were more than 13,000 residential areas in Shanghai and the separation compliance rate increased from 15% (at the end of last year) to 80%. Compared with last October, the city’s recyclables recovery has increased 4.6 times, and wet waste distribution has been doubled [5]. However, this rigid waste separation policy, which is based on the law and emphasizes penalties and constraints, has aroused scholars’ discussion on the sustainability of its impact.

Gaebler et al. [6] pointed out that rigid policies are not conducive to the cultivation of public habits in the long run; on the contrary, it is easy to lead to a backlash of antagonistic behavior after the disappearance of the system. On this basis, a flexible environmental policy, which is opposite to the rigid environmental policy, has been proposed by some scholars. The flexible environmental policies aim to regulate the behavior of enterprises and individuals through market signals, and encourage the public to take the initiative to reduce environmental disruption activities [7,8]. For example, an empirical study on solid waste management in Mumbai suggested that local governments and citizens need to cultivate a sense of common interests and achieve a win–win situation through the public’s initiative separation behavior [9]. Combined with the theory of planned behavior, Zhang et al. [10] analyzed the participation of waste separation through a questionnaire survey of residents in Guangdong of China and pointed out that moral obligation-oriented publicity and advocacy are particularly effective in improving the participation rate of waste separation. Yuan and Zhang [11] found that the flexible environmental policies are significantly less expensive than the traditional environmental policies and are more beneficial to the sustainable development of society. However, the long-term effectiveness of the mandatory separation policy, which is being widely implemented in China, remains to be further explored.

Many scholars have used economic data to assess the impact of policy on urban economy and environment; however, not much of the literature considers the impact of the public’s response on the effectiveness of policy implementation [12]. As the ultimate implementer of policy, the public’s indifference or even resistance to the policy would lead to its unsustainable and ineffective implementation [13]. Therefore, clarifying the characteristics of public response to waste separation policies is an important condition for ensuring policy effectiveness. Arezu and Hamideh [14] pointed out that environmental attitudes can effectively predict individual environmental behavioral performance, and public’s attitudes towards waste separation policies determine whether they will conduct the waste separation behavior. Notably, attitudes include three dimensions, namely, cognition, emotion, and behavior intention [15]. Chang [16] concluded that environmental cognition has a positive facilitative effect on environmental behavior. Additionally, Wang and Jing [17] found that the low-carbon emotion of Chinese urban residents will affect their low-carbon purchasing behavior. Therefore, this study focused on the perspective of policy executors and attempted to analyze the Chinese public’s attitude and response to the rigid and flexible waste separation policies from the perspectives of understanding, willingness to support, and willingness to implement, with the goal of providing a reference for enhancing the effectiveness of the policies.

With regards to the methodological choice, most scholars have used questionnaires when studying the relationship between environmental attitudes and environmental behavior [18]. This approach is also widely used in the analysis of environmental policy responses; especially for the policies that have not been implemented and have less been discussed by the public, the questionnaire survey method of presupposition is often used [19]. Considering that China’s flexible waste separation policy has not been widely implemented, the research on the response characteristics of this policy in our paper also adopts the method of situational survey. That is, we understand the public’s understanding and willingness to support and implement the flexible waste separation policy through the presupposition questionnaires.

In view of the mandatory waste separation policy that has been implemented and widely discussed by the public, this study used big data mining technique to analyze public’s understanding, support willingness, and implement willingness regarding the policy through the big data texts generated by Internet users browsing and discussing this topic. The reason for choosing the method of big data text analysis is as follows. According to the 44th Statistical Report on the Development of China’s Internet published by China Internet Network Information Center (CNNIC) [20], the scale of Chinese Internet users had reached 854 million and the Internet penetration rate had reached 61.2% by June 2019. Huge groups of Internet users are disseminating massive data every day, which may hide a large number of individual information. Using a big data mining technique to obtain the public’s response characteristics, such as the degree of understanding and the willingness to support and implement the Chinese rigid waste separation policy, can not only save time and expand the scope of the survey, but also overcome the influence of the subjects’ vigilance psychology on the analysis results. It is conducive to accurately perceiving the public’s real attitude towards the mandatory waste separation policy [21,22].

**Figure 1:**
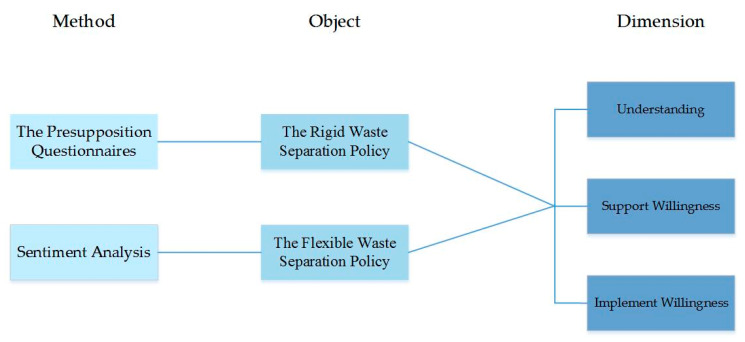
The roadmap of the research.

Based on the above analysis, this paper attempts to study the response characteristics of the Chinese public to waste separation policy from two parts. The first part is a study on the response of the Chinese public to the rigid waste separation policy. We use a big data mining technique to obtain the public’s sentiment text of this policy, and further analyze the response characteristics of the public to the rigid waste separation policy. The second part is a study on the response of the Chinese public to the flexible waste separation policy. We use the situational survey to analyze the public’s potential understanding, support willingness, and implement willingness regarding the policy. The roadmap of the research is shown in Figure 1. We hope this study could provide an important reference for clarifying the long-term effectiveness of China’s mandatory waste separation policy and for formulating a more effective policy to guide waste separation action.

## 2. Study I: The Response Study of Rigid Waste Separation Policy

### 2.1. Research Method Selection and Process Design

With the continuous development of Internet technology, the new generation of information technology, which is represented by “big data mining technique” is widely used in various fields and industries [23]. In the past, big data mining techniques were often used to interpret consumers‘ spending tendencies because information about people’s needs and preferences often hides behind the huge amounts of data, which can help merchants adjust their sales strategies more accurately [24]. In recent years, a growing number of scholars have realized that big data can also help governments understand public demand and preferences for policy. For example, Kim et al. [25] pointed out that governments can improve their service capacity by analyzing big data and can address multiple challenges from transportation, healthcare, etc. Brown et al. [26] also found that governments can make more scientific and accurate policy decisions by making full use of massive data resources. The emergence and widespread use of social networking sites make the dissemination and exchange of emotional information more active than ever before [27], and the emotions in information has been more diverse [28,29]. On the basis of this theory, it has become a popular method to explore public policy issues in academic research by analyzing the text sentiment of the big data obtained from mining and thus obtaining the corresponding conclusions. For example, Jacqueline et al. [22] used the method of big data computational text analysis to explore the level of concern in the community about air pollution issues and related policies in Hong Kong. Therefore, this study will use the big data mining technique to explore public’s response to the rigid waste separation policy in depth through the collection and analysis of relevant information on the web.

The specific research steps are as follows.

1. Use Python to write crawlers, crawl the required data, and carry out simple cleaning and sorting;
2. Use stuttering participles to cut the data and transform the text into independent entries;
3. Analyze the processed text through sentiment analysis, keyword analysis, and other methods, and then draw relevant conclusions.

### 2.2. Data Acquisition and Pre-Processing

According to the 44th Statistical Report on the Development of China’s Internet released by CNNIC [20], the scale of Chinese Internet users had reached 854 million and the Internet penetration rate had reached 61.2% by June 2019. The group of Internet users aged 10–39 accounted for 65.1% of the total number of Internet users by June 2019; the proportion of Internet users aged 40–49 increased from 15.6% (at the end of 2018) to 17.3%; the proportion of Internet users aged 50 and above increased from 12.5% (at the end of 2018) to 13.6%; and the Internet continued to penetrate into the middle and upper age groups. Moreover, the rural population accounts for the largest proportion of people who do not use the Internet. The age, lack of skills, and limited educational level are the main reasons for their non-use of social media [20]. These characteristics imply that individuals in rural areas pay less attention to the environment and are less likely to actively express their views on environmental policies [30]. In China, although some supporters may remain silent on the Internet, there are also some opponents who are unwilling or unaccustomed to express their opinions on the Internet. Therefore, choosing the big data mining technique to analyze the characteristics of individual emotional response is in line with statistical requirements.

In order to ensure the reliability of the data sources, the official mediaPeople’s Daily, the self-media Sina Weibo and the online community Tianya Forum were selected as the data source websites. Considering that the mandatory waste separation started in Shanghai, and Internet users in Shanghai are much more enthusiastic about this event than that in Zhengzhou and Beijing, this study chose “Shanghai waste separation” and related terms as keywords. Given that the Shanghai Municipal Domestic Waste Management Regulations officially came into effect on 1 July 2019, the time period chosen for this study is 1 July 2019 to 31 November 2019, for a total of five months. Subsequently, we used Python to capture more than 16,000 pieces of content, including original microblogs, original posts, and all comments; filtered the noise data using regular expressions; deleted emoji, URLs, and other words. Finally, a total of 10,057 entries about Shanghai waste separation were collected. Among them, 891 entries were derived from People’s Daily, 409 entries were derived from Tianya Forum, and the remaining entries were derived from Sina Weibo. The amount of entries corresponds to the average daily active number of the three Internet platforms.

Notably, the noise-reduced data could not be directly used to sentiment analysis, so the text needed to be subdivided into separate words for the captured words. In this study, the text was processed using stuttering participles, followed by the deletion of deactivating words such as “of”, “this”, “then” to improve the efficiency of the analysis. After cleaning and splitting, the text contained a lot of information, which could be used in the subsequent sentiment analysis and other related research.

### 2.3. Sentiment Analysis

Based on the perspective of “implementation side”, this study investigated public’s response to the rigid waste separation policy in three dimensions (i.e., understanding, support willingness, and implement willingness) through sentiment analysis of the post-processing data.

In this study, text sentiment analysis based on an artificial lexicon was used. Sentiment dictionaries for sentiment analysis have been used extensively in previous research, and the authoritative sentiment dictionaries are the HowNet Sentiment Dictionary, the BosonNLP Sentiment Dictionary, and various sentiment dictionaries that have been improved on this basis. These sentiment dictionaries are developed for a universal research context, and there is a lack of specific sentiment dictionaries applicable to the field of environmental policy. If the existing dictionary is used directly, it may affect the final emotional disposition. For example, the word “rubbish” is a negative word in the general sentiment dictionary, but in the field of environmental policy, especially in the field of policy research on waste separation, it refers to the waste entity itself and should be removed in the analysis. Therefore, in order to improve the accuracy of textual sentiment analysis, this study constructed a dedicated sentiment lexicon dedicated to the study of the waste separation policy for each of the three dimensions of policy understanding, support willingness, and implement willingness.

As a result of the continuous development of Internet technology, cultural communication has become simpler, and some expressions that are not common in the past have become conventional for Internet users and are widely used in Internet interactions, and can express public attitudes more strongly. These words, such as “booboo” (which means a stupid mistake) and “lame” (which means lousy), can easily be overlooked by machines if they are not singled out. Therefore, the sentiment dictionary was mainly composed of two parts, namely, the general sentiment dictionary and the internet term sentiment dictionary. The general sentiment dictionary mainly referred to the HowNet sentiment dictionary, and some out-of-the-ordinary words were deleted manually; the sentiment dictionary of Internet terms referred to the Xinhua Internet Language Dictionary, and a large number of words with personal sentiment that have appeared on the Internet in recent years were added. Finally, the dedicated sentiment dictionary contained a total of 1025 common sentimental words. Because this investigation was carried out in the context of China, the sentiment dictionary was edited in Chinese. In the process of translation, we attempt to clarify the profound meaning of each word and translate it into English properly. The Chinese version is available on request.

To distinguish the strength of words containing sentiment, the strength of words containing sentiment was manually assigned with two negative scores (i.e., a score of “−1” and “−2”) and two positive scores (i.e., a score of “+1” and “+2”). Examples are shown in Table 1.

**Figure 2:**
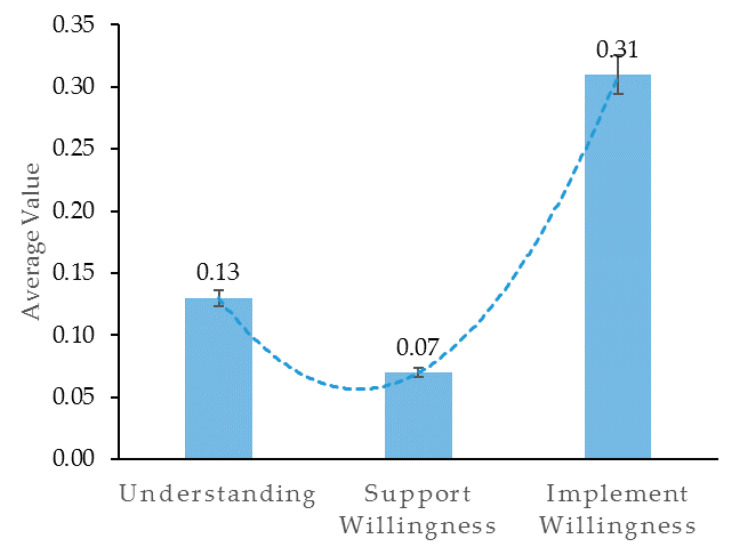
Public’s response to the rigid waste separation policy: understanding-support willingness-implement willingness.

After the construction of the sentiment lexicon was completed, this study used the sentiment lexicon to perform sentiment analysis on the pre-processed text to obtain scores on three dimensions for each phrase grabbed. If a sentence contains multiple sentiment words, the value with the largest absolute value of scores obtained was the final sentiment score for that sentence. Statistical values of sentimental tendencies were taken and the final results are shown in Table 2. The graph based on the data in the Table 2 is shown in Figure 2.

From Figure 2, we observe that the public’s response to the Shanghai waste separation policy can be characterized by a U-shaped change. The mean value of the public’s willingness to support the policy is the lowest (0.070), while the degree of understanding and the willingness to implement the policy is higher, reaching 0.130 and 0.310, respectively. However, from Figure 1 we observe that the public’s understanding, support willingness, and implement willingness regarding the rigid waste separation policy are all positive values, meaning that public’s overall positive sentiment towards the policy is greater than that of negative sentiment.

**Figure 3:**
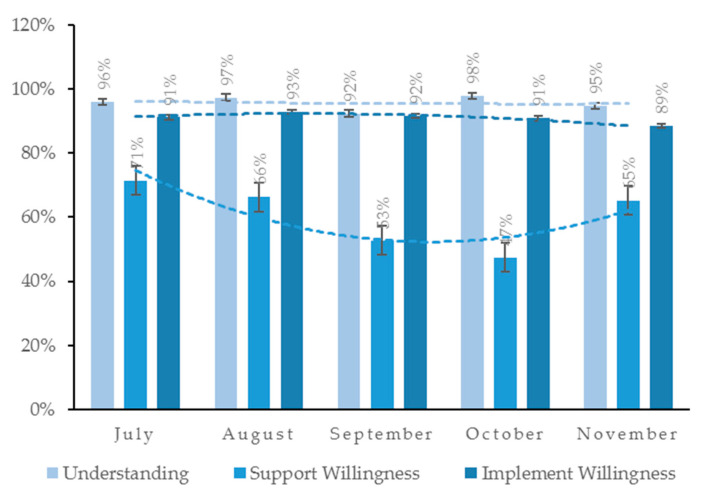
Trends in public’s understanding-support willingness-implement willingness regarding the Shanghai waste separation policy.

In order to investigate the change in public sentiment towards the rigid waste separation policy in the past six months, the study investigated the proportion of positive sentiment in public’s understanding, support willingness, and implement willingness from July to November. The graph is shown in Figure 3.

From Figure 3, it can be seen that public’s understanding regarding the Shanghai waste separation policy remained basically unchanged and kept a high level in the past six months after its formal implementation. The implement willingness has also not changed much, but the overall trend is slightly decreasing. The support willingness slightly increased in November after a significant decrease, but the overall trend is decreasing.

### 2.4. Keyword Analysis

In order to further explore the barriers that may affect public’s willingness to support and implement the rigid waste separation policy, keyword statistics were conducted on the captured texts. After cutting the long text by stuttering, the more frequent words were counted, and these words were analyzed by category to identify the key factors that might affect the final implementation of the rigid waste separation policy. This is helpful to provide some reference and new ideas for the government to formulate or adjust the policy.

First, this study classified all texts into positive sentiment texts, negative sentiment texts, and neutral sentiment texts based on the specific emotion values of the texts, and conducted a corresponding keyword analysis of positive sentiment texts and negative sentiment texts, respectively, with an attempt to identify factors that may facilitate or hinder the implementation of the rigid waste separation policy.

**Figure 4:**
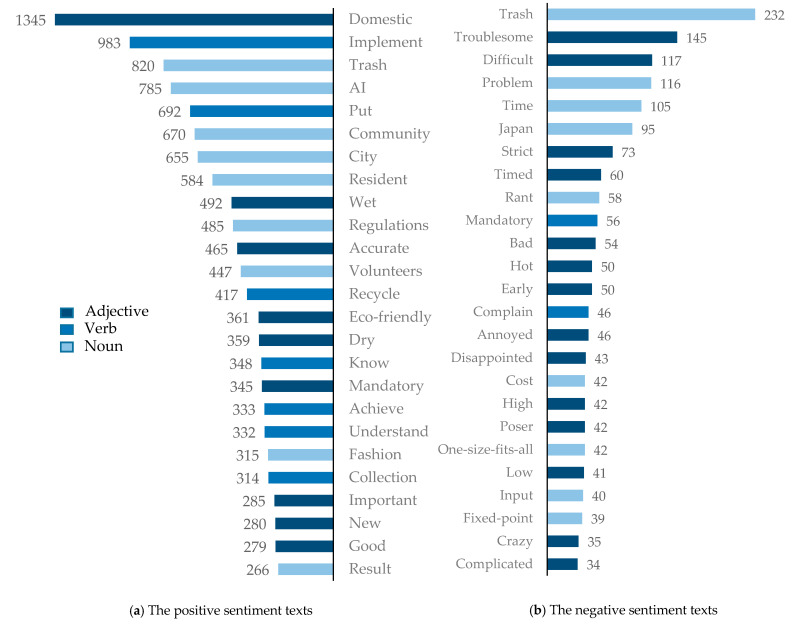
The keywords presentation of Chinese people’s positive and negative sentiment texts to the rigid waste separation policy (partial).

Due to the long text and large number of keywords, the top 25 words with the highest frequency of occurrence were each selected after removing some meaningless or repetitive words. The statistical results are shown in Figure 4.

This study first analyzed the keywords in the positive sentiment text. Adjectives such as “Eco-friendly”, “good”, and “important” appear prolifically in positive sentiment texts, suggesting that public support for the rigid waste separation policy mainly comes from an understanding of its long-term significance. The words “fashion”, “know”, and “understand” indicate that the rigid waste separation policy has been effectively implemented and waste separation has become a fashion. By retracing the position of the words “collection”, “AI”, and “occupation” in the positive sentiment text, we found that when the public is implementing the waste separation policy, some businesses are also seizing business opportunities to create wealth by providing collection services or developing automatic sorting machines for residents who are busy or unwilling to separate the waste. This makes up for the shortcomings of the rigid waste separation policy in terms of time cost and indirectly facilitates the implementation of the rigid waste separation policy.

When capturing keywords in the negative sentiment text, some meaningless and repetitive keywords were removed because the repetition of keywords would cause the barriers to be overlooked. We focused on the keywords that might indicate the reasons that prevented the public from separating waste. As can be seen from Figure 3, the words such as “troublesome”, “annoyed”, “bad”, and “strict” fully reflect the dissatisfaction of the public in implementing the rigid waste separation policy. The words “difficult”, “problem”, and “tangled” indicate that the public does not have a clear understanding of how to separate waste, and there are difficulties in implementing the policy. A review of the placement of the words “timed” and “cost” in the text reveals that the public perceives the implementation of the rigid waste separation policy to be time-consuming and detrimental to the working population. The high frequency of the words “timed”, “one-size-fits-all”, and “fixed point” in the negative sentiment text reflects the public’s doubts and dissatisfaction with the provision of “withdraw the bucket, timed fixed point” in the rigid waste separation policy.

It should be noted that the word “Japan” appears prolifically in the negative sentiment text. Through the keyword positioning of the text, we found that when discussing the waste separation policy in Shanghai, the public habitually compared China’s waste separation policy with Japan’s waste separation policy, and the sentiment was mostly negative. That is, they did not like the design of China’s waste separation policy and preferred Japan’s waste separation policy. Compared to China, Japan’s waste separation was implemented earlier and has a relatively better system (Toshi et al., 2019) [31]. Toshi et al. [31] also pointed out that Japan has a strict legal system that regulates waste disposal, and it adopts a sorting and recycling system similar to China. However, this strict model of restraint and punishment has not led to mass discontent and low willingness to support the policy; conversely, the majority of the public takes the initiative to separate waste and even reports others for violations (Zheng et al., 2017) [32]. This situation is different from the situation of low public support after the implementation of the rigid waste separation policy in China.

**Figure 5:**
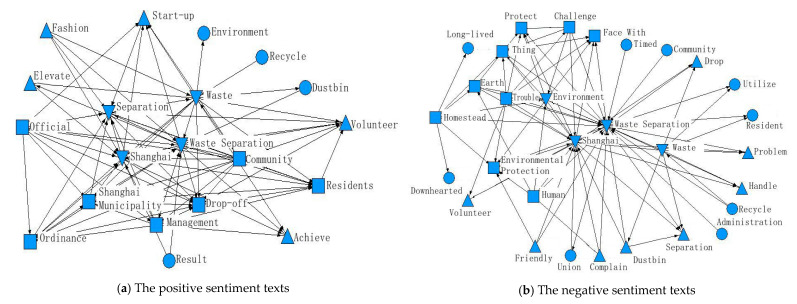
Network relationship diagram of high-frequency keywords for Chinese people’s positive and negative sentiment texts regarding the rigid waste separation policy.

In order to analyze the possible reasons for the low public support of the rigid waste separation policy and to identify the potential barriers, this study used the ROST CM6 “Social and Semantic Networks” section to process the positive and negative sentiment, and thus obtained a high-frequency keyword network relationship diagram (see Figure 5).

There are four layers in each of the two images in Figure 5. In order to distinguish the importance, different graphics were used for identification. Firstly, the correlation of keywords in the positive sentiment text was analyzed, in which the first core layer is the graph of inverted triangle logo. From the collinear relationship, the collinear times of “Shanghai” and “waste separation” are the highest, followed by the collinear times of “separate”, “waste”, and “Shanghai” and “waste separation”. This is mainly due to the keyword setting when collecting data. The second layer is the sub-core layer, i.e., the part represented by the square in the diagram. The high correlation between the words “official” and “ordinance” and the words “Shanghai” and “management” shows that the official regulations on waste separation in Shanghai have effectively raised the positive public sentiment towards waste separation activities. The words “community”, “residents”, and “drop-off” indicate that community waste separation is the most important part of public waste separation. The third layer is marked by a triangle, and the words “elevate” and “fashion” are highly related to the keywords that in the first two layers, indicating that waste separation activities have become the new fashion and have increased public satisfaction, and the existence of “volunteer” has also effectively promoted the implementation of waste separation policies. The fourth layer is the peripheral layer, i.e., the part marked by small dots. This part of words has less collinear times with the main keywords, but it can also influence public sentiment towards the rigid waste separation policy, such as the process of recycling and management of waste and the time cost of waste separation.

The same pictorial identification rules were applied to the results of the analysis of negative sentiment texts. The first core layer of words is similar to the results of the positive sentiment text, i.e., the words like “waste separation”. In the sub-core layer, the words “trouble” and “challenge” are highly related to the words “environmental protection”, “earth”, “homestead” and other words, indicating that the public has recognized the importance of waste separation to environmental protection. However, the “trouble” of waste separation hinders the generation of separation behavior, which is also the main reason for the public to have more negative sentiment towards the rigid waste separation policy. In the sub-peripheral layer marked by a triangle, there are more collinear times regarding the three words (“complain”, “drop”, and “dustbin”) and the word “waste separation” in the core layer, indicating that the public has more complaints about waste separation activities, and the drop process also leads to more negative public sentiment. The fourth layer is the peripheral layer, i.e., the part marked by small dots. This group of words has less collinearity with the main keyword, but can also lead to negative public sentiment towards the rigid waste separation policy, such as waste recycling efforts and the effectiveness of waste separation.

Based on the above analysis and combined with the query of the relevant contents of the Shanghai Domestic Waste Management Regulations, this study concluded that a clear waste separation policy is conducive to increasing public support. The situation that waste separation has become a fashionable behavior advocated by society and the participation of volunteers are also an important reason for the public to show more positive sentiment towards the rigid waste separation policy. An analysis of negative sentiment texts found that “troublesome” was the main reason that prevented the public from separating waste, and the process of placing waste is the main source of negative sentiment among the public.

## 3. Study II: The Response Study of Flexible Waste Separation Policy

### 3.1. Scale Design

In recent years, with the development of the flexible management theory, its application in the field of environmental policy has also attracted many scholars’ attention. Contrary to the rigid environmental policy, the flexible environmental policy attaches more importance to regulating the behavior of enterprise and individual through market signals [7,8]. Taking the environmental deposit system as an example, consumers need to pay an additional amount of recycling deposit when purchasing a potentially contaminated product; when the product’s life cycle ends, consumers need to return the waste product to the designated recycling channel before the deposit can be returned [33]. Such flexible environmental policies have been widely applied in Japan, Sweden, Canada, and other countries, and have been proved to have an important effect on reducing waste production at the source [34]. In order to investigate whether the flexible waste separation policy can achieve similar effects in China, this study further explored the Chinese public’s understanding, support willingness, and implement willingness regarding the flexible waste separation policy. Considering that the flexible waste separation policy has not been widely applied in China, the visibility and discussion on the Internet are obviously insufficient. If we use a big data mining technique to collect data, the total amount of data will be relatively small, leading to the inability to accurately perceive public attitudes. Therefore, this study used the survey method of presupposition scenario. That is, the government was assumed to have implemented one of the flexible waste separation policy in the questionnaire; by explaining this policy to the public, we inquired about public’s understanding, support willingness, and implement willingness regarding the flexible waste separation policy in the hypothetical scenario.

Through reviewing and sorting the current waste separation policies in the world, the relevant policies in line with flexible management were selected, including deposit return policy, waste separation scheme, credit system, etc. The initial scale was obtained by following the existing mature scales related to policy response [12,19], combined with the actual situation, supplemented by methods such as expert interviews to revise and develop relevant measurement questions. To test the feasibility of the scale, a pre-survey was conducted from 10, August, 2019 to 25, August, 2019 and 121 valid questionnaires were collected.

The pre-test questionnaire was tested for reliability and validity, and the questions were corrected to form a formal questionnaire. The final questionnaire was divided into two parts. The first part was a basic household information survey, which mainly included the number of household members in the sample, monthly household income, etc. The second part was a situational simulation survey, in which the public was asked to pre-determine the implementation of a specific waste separation policy and to rate their understanding, support willingness, and implement willingness. Example items for the volume representation are shown in Table 3.

### 3.2. Formal Research and the Testing for Reliability and Validity

After the formal scale was obtained, the study began the formal research. The first step was to determine the sample population for the research. Since waste separation was mainly carried out by households, this study collected questionnaires by households, and distributed the sample structure in reasonable intervals regarding age, monthly income and so on through stratified sampling, so as to ensure the representativeness and scientificity of the sample. The official questionnaire was given out during the period of 19, September, 2019 and 22, October, 2019 in Xuzhou, Suzhou and other cities of Jiangsu Province. The way of visiting residential areas was adopted. Finally, a total of 325 questionnaires were gave out during the on-site survey, and 279 valid questionnaires were collected, with an effective recovery rate of 85.84%.

In this study, the reliability and validity of the questionnaire were examined by using AMOS 17.0 and SPSS 21.0. With regard to the structural validity of the questionnaire, the fit indicators obtained by validation factor analysis were: χ^2^ = 1088.393, df = 341, χ^2^/df = 3.192, RMSEA = 0.050, GFI = 0.912, IFI = 0.900, CFI = 0.900, TLI = 0.889, implying that all indicators are in good range. For the reliability test of the sample, the results showed that the Cronbach’s Alpha coefficient was greater than 0.7 for all three dimensions, indicating that the data obtained by the scale has a good internal consistency.

### 3.3. Result Analysis

Statistical analysis of the data can reveal the sentimental tendency of public’s understanding and their willingness to support and implement regarding the flexible waste separation policy. The details of the analysis are shown in Table 4.

**Figure 6:**
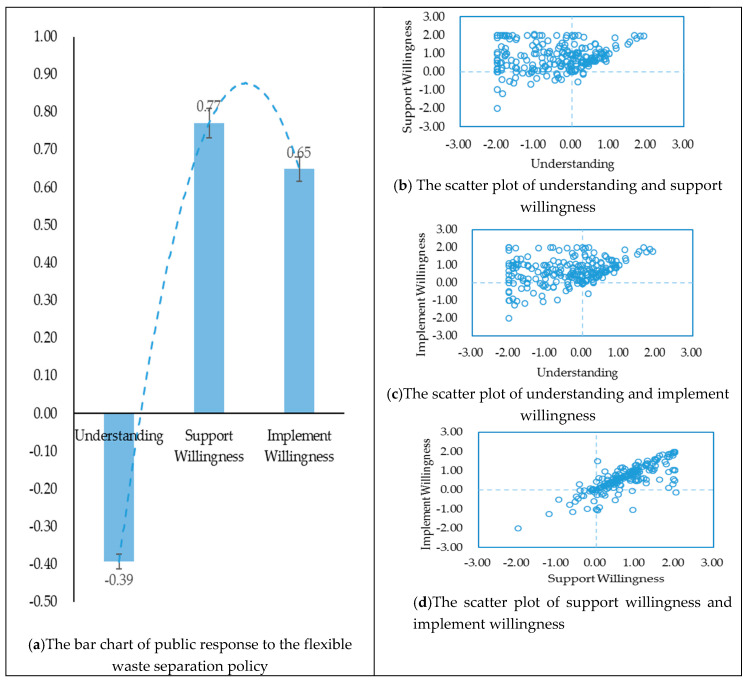
Public’s response to the flexible waste separation policy: understanding-support willingness-implement willingness.

Based on the data in Table 4, the corresponding bar and scatter plots were drawn in this study (see Figure 6).

From the bar graph in Figure 6, it can be seen that public’s response to the flexible waste separation policy presents an inverted U-shape change. That is, the score of understanding is negative and low (−0.39), while the scores of support willingness and the implement willingness are both positive (0.77 and 0.65, respectively). In other words, the public’s willingness to support the flexible waste separation policy is the highest; the willingness to implement the policy is lower than the willingness to support it, but it is still a positive value; the understanding of the policy is the lowest and its mean value is negative, indicating that the majority of the respondents are not clear about the specific contents of the flexible waste separation policy.

From the scatter plot in Figure 6, it can be seen that the datapoints are mainly distributed in the first and second quadrants in the scatterplot with the X-axis of policy understanding and the Y-axis of support and implementation, respectively. The results indicate that public’s understanding regarding the flexible waste separation policy varies greatly, while the support and implement of the policy are concentrated in positive attitude. The datapoints for policy support willingness and policy implement willingness are mainly distributed in the first quadrant and approximated as a straight line through the origin. This suggests that the public who exhibits a higher willingness to support the flexible waste separation policy also tends to have a higher willingness to implement the policy.

## 4. Discussion

The results of data analysis show that the public’s understanding, support willingness, and implement willingness regarding the mandatory waste separation policy are all positive values, and public’s responses present a U-shaped change. This suggests that the public has a high level of understanding and willingness to implement the rigid waste separation policy; however, the public’s wiliness to support this rigid policy is not high. In general, individuals who pay attention to the waste separation policies are more likely to understand how to separate the waste and tend to put it into practice more easily [35]. Although the lack of support for the policy itself will reduce public’s willingness to separate waste, the punishment and restriction mechanisms of the rigid waste policy can make the rational public choose to avoid the risk, that is, to choose to engage in waste separation activities [36]. Notably, this forced choice can lead to an increase in negative public sentiments such as dissatisfaction and resistance, leading to a lower level of public support for the rigid waste separation policy. Of course, there are also some individuals who have a particularly strong sense of environmental protection and social responsibility. They tend to choose to implement the rigid waste separation policy that is conducive to environmental protection, even if this may damage their own benefits [37,38]. In addition, the deteriorating environment and the awareness of environmental protection will also improve the public’s attitudes towards environmental policies [39], which means that the public shows a positive attitude towards the rigid waste separation policy.

The results of the keyword analysis show that a clear policy on waste separation is conducive to increasing public support. Chukwuka et al. [40] pointed out that the lack of practical policy benchmarks would affect the effectiveness of environmental policy implementation. This may be due to the uncertainty of the policy, which leads to different public understanding of the policy and ultimately leads to more confused emotions being expressed in the implementation of the policy. Moreover, the participation of a volunteer team and the advocacy of the whole society can improve the subjective well-being of the environmentally followers [41], and also can provide assistance to individuals with confusion, which is conducive to the generation of environmental protection behavior.

The study on the flexible waste separation policy shows that the public’s understanding-support willingness-implement willingness regarding the flexible waste separation policy presents an inverted U-shaped change. That is, the public’s willingness to support the rigid waste separation policy is high, but the degree of policy understanding is low. In other words, the public is very supportive of the flexible waste separation policy and is willing to implement it, but they show a lower level of understanding when implementing the specific policy standards. The scatter plots for the three dimensions also verify this result. The public’s understanding of the policy is quite different, but there are more positive emotions in the willingness to support and the willingness to implement, and the public with higher support willingness tends to show a higher implement. This may be due to the lack of promotion of the Chinese government’s incentive policies in flexible waste separation management and the fact that the public has a single channel to understand public policy, resulting in a lack of in-depth public awareness and understanding of the flexible waste separation policy. Although the degree of public’s policy understanding is very low, when the public is informed of the general content of the flexible waste separation policy through the questionnaire, they tend to show a high willingness to support and implement. This may be that the respondents perceived the environmental benefits of flexible waste separation management, and thus formed a positive attitude towards waste separation [42].

Compared to the flexible waste separation policy, the degree of public’s understanding regarding the rigid waste separation policy is higher. This may be due to the fact that the large-scale discussions on the Internet caused by the rigid waste separation policy have increased the possibility of public access to the specific content of the policy, and the policy’s emphasis on punishment mechanisms has motivated the public to take the initiative to understand how to separate waste. However, public’s willingness to support the rigid waste separation policy and its implementation is lower than that of the flexible waste separation policy. That is, a portion of the public neither supports the rigid waste separation policy nor is willing to carry out waste separation. This may be due to the fact that negative sentiment such as “troublesome” and “annoyed” inhibit public’s willingness to act, while lower willingness to act leads to less actual behavior [43]. Additionally, this study also analyzed the trend graph of public response to the rigid waste separation policy. The results showed that in the nearly six months after the implementation of the rigid policy, public’s understanding regarding the policy did not change much; the willingness to implement the policy slightly decreased; and the overall willingness to support the policy showed a decreasing trend. In other words, the public increasingly did not support the rigid waste separation policy over time. Most people were forced by the system to still carry out waste separation, but a few people were unwilling to implement this policy. The negative sentiment and willingness may be due to the fact that the public gradually perceives that certain inconveniences associated with rigid waste separation (e.g., high time costs) during the policy implementation; such loss of for some individuals is greater than the loss of money resulting from not separating waste, which makes the public prefer the penalty of not separating to segregating [44,45].

It should be noted that, in the keyword analysis of the negative sentiment text of the rigid waste separation policy, the public was observe to have more positive sentiment towards the Japanese waste separation policy compared to the Chinese waste separation policy. Although the restriction and punishment of waste separation policy in Japan is more strict than that in China, the support rate of the Japanese public for the policy is relatively high, and they are willing to take the initiative to separate waste [32]. This may be due to the fact that Japan pays more attention to the cultivation of waste separation habits and public social responsibility; they consider waste separation as an individual social obligation and integrate it into the education of the public, especially the youth group [46]. Such policies that favor flexible management have achieved better results than rigid policies in cultivating the active public separation behavior.

## 5. Conclusions

In this paper, we focused on the perspective of policy executor and explored the Chinese public’s attitude and response to the rigid and flexible waste separation policies from the perspectives of understanding, willingness to support, and willingness to implement. The study on the rigid waste separation policy showed that “public’s understanding-support willingness-implement willingness” regarding waste separation policy was characterized by a U-shaped response. That is, public’s understanding and willingness to implement the mandatory waste separation policy were relatively high in the short term, but the willingness to support this policy was relatively low. The monthly statistics on public’s response to the rigid waste separation policy showed that the public’s understanding of the policy did not change much over time, but the willingness to support and implement showed a downward trend over time. Through the analysis of the key words regarding the reasons for the decline in the public’s willingness to support and implement waste separation activities, we found that the “troublesome” implementation of separating the waste is the most paramount reason preventing the public from carrying out the waste separation activities, and the promotion activities about the waste separation was the main source of the negative sentiment. The results imply that a clear waste separation policy is conducive to increasing the public’s willingness to support the policies, and waste separation has become a fashionable behavior advocated by society. The participation of volunteers are also important factors for the public to show more positive attitudes towards the rigid waste separation policy.

Contrary to the response characteristics of the mandatory waste separation policy, the results showed that “public’s understanding-support willingness-implement willingness” regarding flexible waste separation policy was characterized by an inverted U-shaped response. That is, the Chinese public showed a higher willingness to support the flexible waste separation policy, but showed a lower understanding of the policy. Additionally, the scatter plots for each of the three dimensions showed that the public’s understanding regarding the flexible waste separation policy varied among individuals, but the public was observed to have more positive sentiment regarding the willingness to support and the willingness to implement. Particularly, the public with a higher willingness to support was observed to have a higher willingness to implement.

## Figures and Tables

**Table 1:** Examples of Sentiment Dictionaries.

Score	Understanding	Support Willingness	Implement Willingness
+2	unambiguous; absolutely clear; comprehend; Understand	with admiration for; enjoy popular confidence	persist in; unremittingly; persistent; insistent
+1	know; know of; hear of; know about	agree; consent; approve; approve of	try; comply with; abide by; attempt
−1	unknown; unclear; uncertain; indefinite	Unsupported; disapproving; deprecated	hasty; cursory; turn a blind eye; unwilling
−2	ambiguous; complex; never heard; know nothing about	terrible; awful; horrible; dreadful	give up; back-out; reluctance

**Table 2:** Sentimental Tendency.

	N	Mean Value	Standard Deviation	Variance	Skewness		Kurtosis	
	Statistic	Statistic	Statistic	Statistic	Statistic	Standard Error	Statistic	Standard Error
Understanding	10,057	0.130	0.412	0.170	2.493	0.024	9.030	0.049
Support Willingness	10,057	0.070	0.621	0.386	0.393	0.024	4.971	0.049
Implement Willingness	10,057	0.310	0.726	0.527	1.141	0.024	1.644	0.049

**Table 3:** Examples of scale.

Policy Content	Measurement Content	Item Description	Not Conformed	Little Conformed	Common	Conformed	Quite Conformed
“Deposit return policy”: consumers pay a deposit of a certain amount when purchasing a product, which will be returned after the return of used products or recycling of used products.	Understanding	I understand the specifics of this policy standard	−2	−1	0	1	2
	Support Willingness	I support this policy standard	−2	−1	0	1	2
	Implement Willingness	I will obey or abide by the standards of the system	−2	−1	0	1	2
		I will make this policy standard known to others and drive more people to follow it	−2	−1	0	1	2

**Table 4:** Sentiment disposition statistics.

	N	Mean Value	Standard Deviation	Variance	Skewness		Kurtosis	
	Statistic	Statistic	Statistic	Statistic	Statistic	Standard Error	Statistic	Standard Error
Understanding	279	−0.392	0.972	0.991	−0.120	0.164	−0.828	0.327
Support Willingness	279	0.771	0.621	0.725	−0.128	0.164	0.194	0.327
Implement Willingness	279	0.649	0.726	0.727	−0.304	0.164	0.518	0.327
